# Differential Expression Analysis by RNA-Seq Reveals Perturbations in the Platelet mRNA Transcriptome Triggered by Pathogen Reduction Systems

**DOI:** 10.1371/journal.pone.0133070

**Published:** 2015-07-14

**Authors:** Abdimajid Osman, Walter E. Hitzler, Adam Ameur, Patrick Provost

**Affiliations:** 1 Department of Clinical Chemistry, Region Östergötland, Ingång 64, Linköping, Sweden; 2 Department of Clinical and Experimental Medicine, University of Linköping, Linköping, Sweden; 3 Transfusion Center, University Medical Center of the Johannes Gutenberg University Mainz, Hochhaus Augustusplatz, Mainz, Germany; 4 Department of Immunology, Genetics and Pathology, Science for Life Laboratory, Uppsala, Uppsala University, Uppsala, Sweden; 5 Université Laval CHUQ Research Center / CHUL 2705 Blvd Laurier, Quebec, QC, Canada; Laboratoire de Biologie du Développement de Villefranche-sur-Mer, FRANCE

## Abstract

Platelet concentrates (PCs) are prepared at blood banks for transfusion to patients in certain clinical conditions associated with a low platelet count. To prevent transfusion-transmitted infections via PCs, different pathogen reduction (PR) systems have been developed that inactivate the nucleic acids of contaminating pathogens by chemical cross-linking, a mechanism that may also affect platelets’ nucleic acids. We previously reported that treatment of stored platelets with the PR system Intercept significantly reduced the level of half of the microRNAs that were monitored, induced platelet activation and compromised the platelet response to physiological agonists. Using genome-wide differential expression (DE) RNA sequencing (RNA-Seq), we now report that Intercept markedly perturbs the mRNA transcriptome of human platelets and alters the expression level of >800 mRNAs (P<0.05) compared to other PR systems and control platelets. Of these, 400 genes were deregulated with DE corresponding to fold changes (FC) ≥2. At the p-value < 0.001, as many as 147 genes were deregulated by ≥ 2-fold in Intercept-treated platelets, compared to none in the other groups. Finally, integrated analysis combining expression data for microRNA (miRNA) and mRNA, and involving prediction of miRNA-mRNA interactions, disclosed several positive and inverse correlations between miRNAs and mRNAs in stored platelets. In conclusion, this study demonstrates that Intercept markedly deregulates the platelet mRNA transcriptome, concomitant with reduced levels of mRNA-regulatory miRNAs. These findings should enlighten authorities worldwide when considering the implementation of PR systems, that target nucleic acids and are not specific to pathogens, for the management of blood products.

## Introduction

Circulating blood platelets play a central role in diverse physiological processes including hemostasis, fibrinolysis, blood vessel repair and inflammation. Derived from their bone marrow precursor megakaryocytes, platelets are anucleate and are deprived of genomic DNA, so they are incapable of achieving de novo genomic DNA transcription. Platelets nevertheless harbor a diverse and functional transcriptome, and their aptitude to carry complex molecular processes, including messenger RNA (mRNA) splicing and translation, is relatively well accepted [1–5].

A platelet count lower than 100 × 10^9^/L is internationally recognized as the threshold for diagnosis of thrombocytopenia and is associated with increased risk of bleeding [6]. This clinical condition can be treated by transfusion of platelet concentrates (PCs) prepared from the blood of healthy donors by blood banks, where they are stored at ambient temperature for several days prior to transfusion. Different pathogen reduction (PR) systems have been developed to prolong the shelf-life of stored blood components, such as PCs, and to prevent transfusion-transmitted infections. PR systems are typically designed to inactivate the nucleic acids of contaminating pathogens through chemical cross-linking mechanisms [7] and their effectiveness in reducing pathogens is acknowledged. However, serious concerns related to the safety of the incumbent PR systems have been raised, particularly their negative impact on platelet viability, as reported by independent clinical studies demonstrating that PR systems cause reduced platelet dose per component as well as significant increase in bleeding in recipients of pathogen-reduced (versus non-pathogen-reduced) platelets [8–10]. The exact mechanisms of these adverse effects are not fully understood. We recently reported that PR treatment negatively affects the level of some nucleic acids in platelets [11]. In that study, we studied the effects of three different PR strategies on stored PCs (gamma irradiation, Mirasol or Intercept), and compared them with their related control PCs, either left untreated or incubated in additive solution. We found that platelets treated with Intercept (amotosalen + ultraviolet-A [UVA] light) exhibited significantly reduced levels of 6 of the 11 microRNAs, and of 2 of the 3 anti-apoptotic mRNAs, that were analyzed by quantitative real-time PCR (qPCR) [11]. We proposed that Intercept-treatment might activate platelets and induce the release of nucleic acids from platelets, and thus impoverish platelets in microRNAs.

These results prompted us to expand our investigation and to document the effects of PR systems on the whole mRNA transcriptome of stored PCs. To this end, we have utilized the Ion AmpliSeq technology in combination with next-generation sequencing (NGS) to explore the differential RNA-expression (DE) of human RefSeq genes (UCSC RefGene) in stored platelets subjected to five different conditions (one control and four treatment groups). Ion AmpliSeq is a new technology based on massively parallel multiplexing PCR used for RNA sequencing (RNA-Seq) of the human transcriptome. The advantage of this technology, compared with other RNA-Seq techniques, is that it provides a direct mRNA quantification and does not rely on normalizations such as FPKM (Fragments Per Kilobase of exon per Million fragments mapped), which is an advantage for DE analysis [12]. The results that we obtained from this comprehensive and detailed RNA-Seq analysis transposed the adverse effects of Intercept-treatment, that we initially documented for selected platelet RNAs, to the whole platelet mRNA transcriptome and indicate that the transcriptome of stored platelets is markedly altered by treatment with Intercept.

## Materials and Methods

### Ethical statement

The study was conducted following the German pharmaceutical law for assessment of the quality of platelet products produced for routine use in hemotherapy and was approved by the Ethics Committee of the Medical Association of Rheinland-Pfalz (Ethik-Kommission bei der Landesärztekammer Rheinland-Pfalz–EK LÄK RLP). All platelet donors gave written informed consent to participate in the study.

### Samples

The study was designed as previously described [11]. Briefly, PCs were prepared from blood donors by apheresis using a standard blood bank protocol [13]. PCs were subjected to one of the following five conditions: (1) control (platelets stored in donor plasma); (2) additive solution (platelets stored in 65% storage solution for platelets [SSP+; MacoPharma] and 35% donor plasma); (3) Irradiation (platelets treated with gamma irradiation [30 Gy] and stored in donor plasma); (4) Mirasol (platelets stored in donor plasma and treated with riboflavin and ultraviolet-B (UVB) light); or (5) Intercept (platelets stored in SSP+–the same medium as in the additive solution–and treated with amotosalen and UVA light). For each group, 4 PC samples were analyzed (n = 4 PCs per treatment, 20 samples in total). Gender distribution was 3 males and 1 female (control), 2 males and 2 females (Irradiation), 3 males and 1 female (SSP+), 2 males and 2 females (Mirasol) and 4 males (Intercept). All PR treatments were performed according to the standard blood bank procedures or the manufacturer’s instructions without modification. RNA was extracted one day after platelet treatment.

### Platelet preparation and RNA extraction

Platelets were isolated from the PCs as previously described [14] minimizing the number of contaminating leukocytes by employing anti-CD45 magnetic beads. The protocol that we have used routinely yield platelets that contain less than 1 leukocyte per 3.2 million platelets [14]. Considering that leukocytes have ~1,000 times more RNA than platelets [15], we calculated that contaminating leukocyte RNA may represent ~0.03% of the platelet RNA preparations, which we consider as negligible. Total RNA was isolated using miRCURY RNA Isolation Kits—Cell & Plant according to the manufacturer’s instructions (Exiqon A/S, Vedbaek, Denmark). RNA quality prior to sequencing was assessed with Quantus Fluorometer (Promega Corporation, Madison, USA) and with Agilent 2100 Bioanalyzer (Agilent Technologies, Palo Alto, Ca, USA).

### RNA-sequencing

Platelet RNA-Seq analysis was performed on an Ion Proton System for next-generation sequencing (Life Technologies, Carlsbad, CA, USA). For each of the 20 samples, 10 ng of total RNA was reverse transcribed using the Ion AmpliSeq Transcriptome Human Gene Expression kit (Revision A.0) following the protocol of the manufacturer (Life Technologies). The cDNA was amplified using Ion AmpliSeq Transcriptome Human Gene Expression core panel (Life Technologies) and the primer sequences were then partially digested. This was followed by ligation of adaptors (Ion P1 Adapter and Ion Xpress Barcode Adapter; Life Technologies), purification of adaptor ligated amplicons using Agencourt AMPure XP reagent (Beckman Coulter Inc., Indianapolis, IN, USA), elution in amplification mix (Platinum PCR SuperMix High Fidelity and Library Amplification Primer Mix, Life Technologies) and amplification. Size-selection and purification was conducted using the Agencourt AMPure XP reagent (Beckman Coulter). The amplicons were quantified using fragment analyzer instrument with the DNF-474 High Sensitivity NGS Fragment Analysis Kit (Advanced Analytical Technologies, Inc., Ames, IA) before the samples were pooled in sets of five. Emulsion PCR was carried out on the Ion OneTouch 2 system with Ion PI Template OT2 200 Kit v3 chemistry (Life Technologies). Enrichment was performed using the Ion OneTouch ES (Life Technologies). Samples were loaded on an Ion PI chip Kit v2 and sequenced on the Ion Proton System using Ion PI Sequencing 200 Kit v3 chemistry (200 bp read length; Life Technologies).

### Analysis of sequence reads and differential gene expression

The Ion Proton reads were analyzed using the AmpliSeqRNA analysis plugin, v4.2.1, in the Torrent Suite Software (Life Technologies). This program counts the number of sequences obtained for all cDNA amplicons. The resulting counts represent the gene expression levels for over 20,800 different genes present in the AmpliSeq Human Gene Expression panel. The expression level counts for all of the 20 different samples were then merged into one single table, and the resulting table was then used for differential gene expression analysis with the R/Bioconductor package DESeq (http://www.bioconductor.org/). The DESeq analysis was performed using standard parameters. Adjusted p-values (padj) for multiple testing, using Benjamini-Hochberg to estimate the false discovery rate (FDR), were calculated for final estimation of DE significance. The GENE-E software, v3.0.2, was used for cluster analysis (http://www.broadinstitute.org/cancer/software/GENE-E/).

### Analysis of microRNA-mRNA correlations

Correlations between microRNA (miRNA) and mRNA levels were performed by using the MAGIA analysis tool [16] with the expression data obtained from differentially expressed genes as well as from 11 microRNAs previously analyzed in the same samples [11]. miRNA target prediction with miRanda (http://www.microrna.org) and PITA (http://genie.weizmann.ac.il/pubs/mir07/mir07_data.html) tools were combined with integrative analysis of miRNA and mRNA expression profiles followed by non-parametric Spearman correlations. Regression analysis was carried out using RStudio software (http://www.rstudio.com/). Network and enrichment analysis were performed on the Cytoscape software v3.2. (http://www.cytoscape.org).

### Submission of the sequencing data to public repository

The complete mRNA expression data for all samples has been deposited to the European Nucleotide Archive (https://www.ebi.ac.uk/ena) and is accessible under the accession number PRJEB8213.

## Results

### Sequencing metrics

A total number of 20 independent platelet samples (4 samples/group) were subjected to RNA-Seq. Targeted sequencing of over 20,800 genes generated a total number of 33 gigabases (mean: 1.6 gigabases/sample). This included all known RefSeq genes. Mitochondrial RNAs as well as noncoding RNAs were not included in the Ion AmpliSeq Transcriptome Human Gene Expression panel used in this study. With the predicted quality of >Q20, the usable sequences corresponded to 29 gigabases (89%) and amounted to a total of 312 million filter pass reads, which were aligned to the human reference genome (GRCh37, assembly hg19). The mean read length was 105 base pairs (bp). The alignment performance at 100 bp reads corresponded to >99% accuracy (AQ20) indicating a satisfactory quality. Mean Coverage Depth ranged 97–109 bp (AQ20) and 85–96 bp (Perfect), respectively. To filter out low-abundant transcripts and achieve greater certainty in differential mRNA expression analysis, we considered only those transcripts whose expression levels corresponded to >1/10,000 of ß-actin’s expression. This stringent threshold brings all transcripts within approximately 13 PCR cycles of ß-actin, as previously described [17].

### The platelet transcriptome at a glance

Mean expression of the 50 most abundant platelet mRNAs in each group is shown in S1 Table. The mRNA profile of the control platelets largely confirmed the transcriptional profile of the most abundant genes in human platelets that was reported by other groups [18–21]. The most abundant platelet mRNA in the control group was thymosin beta 4, X-linked (TMSB4X), consistent with the NGS report of Kissopoulou et al. [19] as well as the microarray and SAGE analysis of Gnatenko et al. [20]. Remarkably, TMSB4X was not the most expressed mRNA in any of the treated groups.

**Fig 1:**
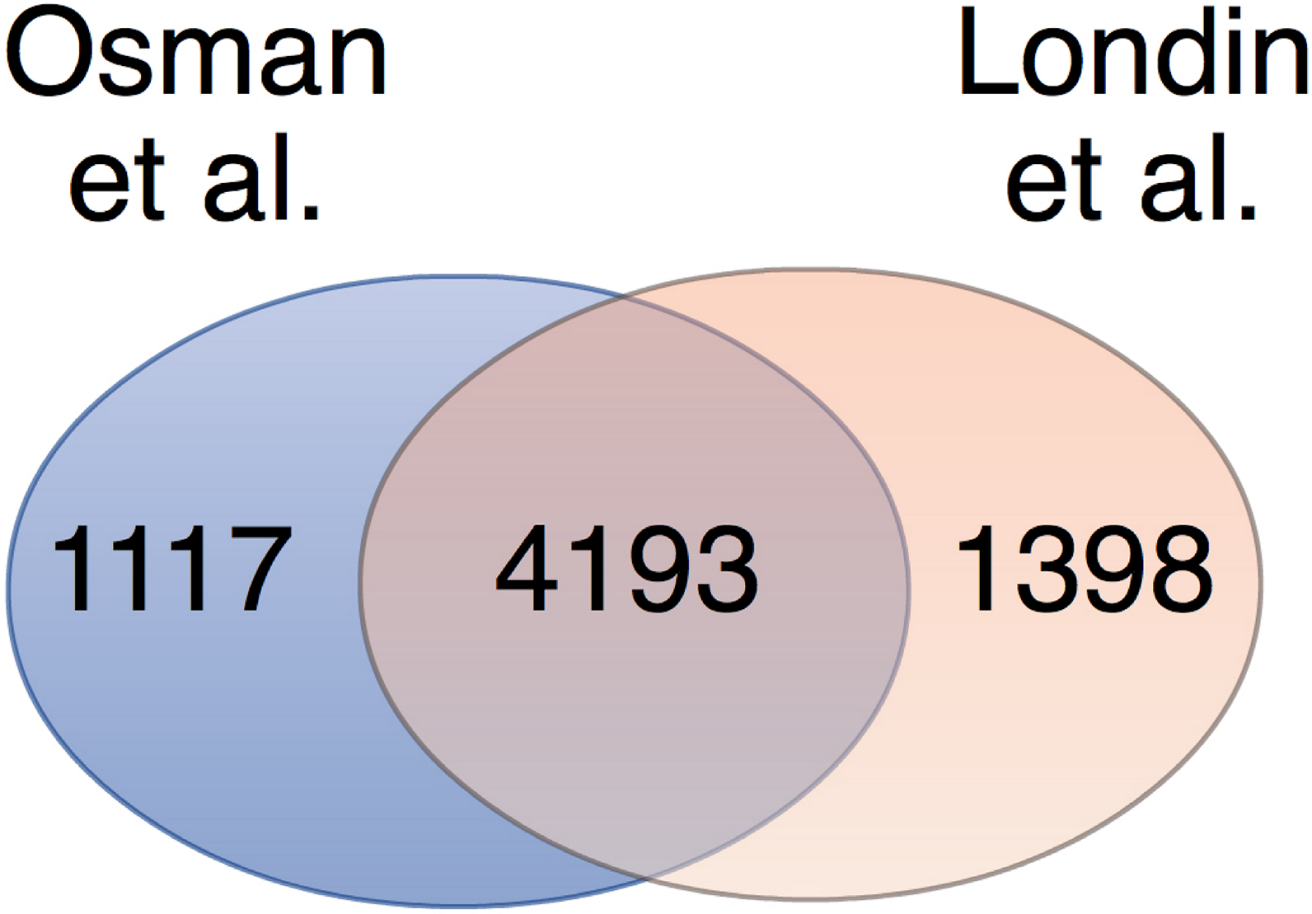
Venn diagram depicting the overlap of detected mRNAs in this study (Osman et al.) with that of Londin et al. [17] are shown. Genes detected above the threshold >1/10,000 of ß-actin expression (this study) or ≥1/10,000 of ß-actin expression (Londin et al.) were compared.

We also compared our mRNA list for the control group with that of Londin et al. [17] who recently reported a list of mRNAs identified to be very highly correlated in platelets of 10 donors. Comparison of the two datasets, both involving threshold setting at ≥1/10,000 of ß-actin expression, revealed that nearly 80% (4193) of all of our detected mRNAs are also found in the list of Londin et al. [17] (Fig 1). This suggested that both datasets correlated fairly well despite that Londin et al. employed total RNA sequencing without rRNA depletion, whereas the present study used the Ion AmpliSeq Transcriptome Human Gene Expression panel containing the RefSeq mRNAs.

**Fig 2:**
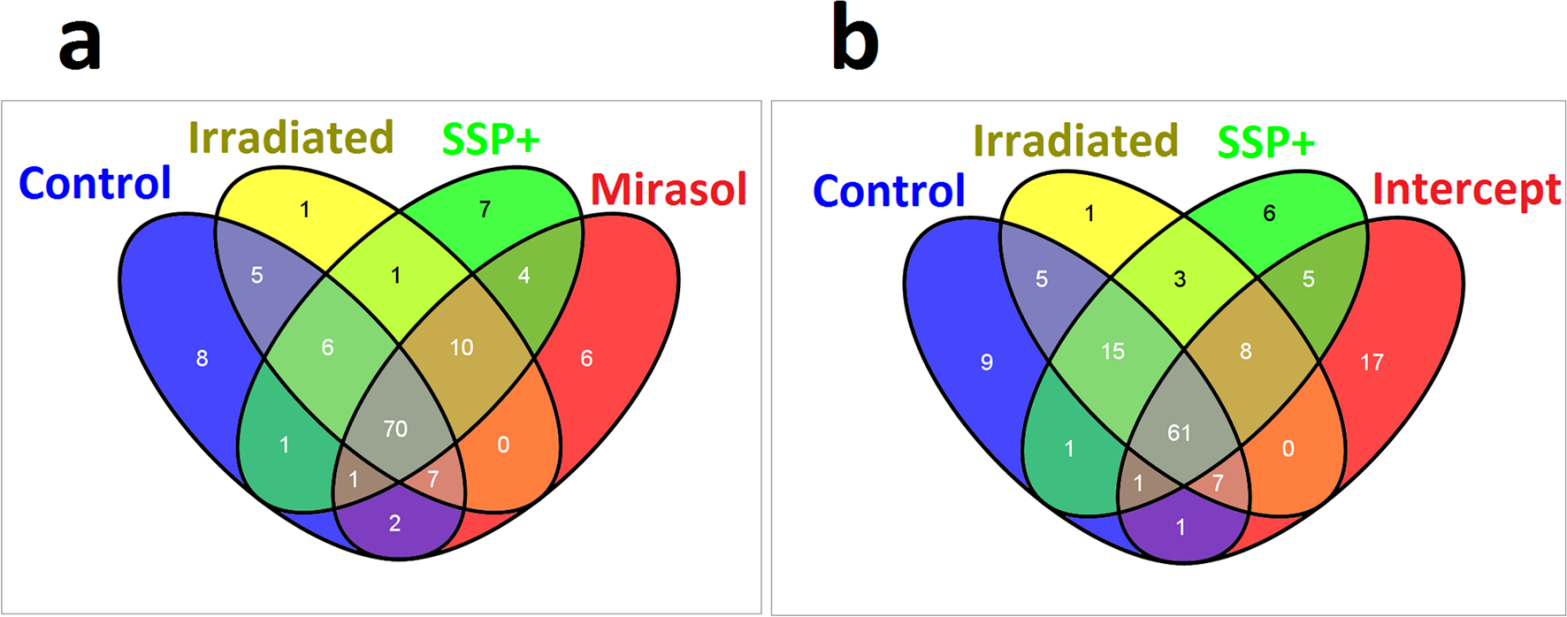
Venn diagrams showing overlap of the top 100 platelet mRNAs in the different platelet groups. a) All groups except Intercept, b) All groups except Mirasol.

In Fig 2, a Venn diagram depicts how the top 100 platelet mRNAs overlap among the different groups. The two most recent PR systems, Mirasol (Fig 2A) and Intercept (Fig 2B), are shown separately for the sake of clarity. The control, Irradiated, SSP+ and Mirasol treated groups had as many as 70 of their top 100 mRNAs in common (Fig 2A). When the comparison included Intercept-treated platelets, instead of Mirasol, this overlap was reduced to 61 genes (Fig 2B). In fact, as many as 23 mRNAs were different in the top 100 genes between Mirasol and Intercept-treated platelets (data not shown), indicating discrepancies in their mRNA profile and suggesting that Mirasol and Intercept may affect the platelet transcriptome differently.

**Fig 3:**
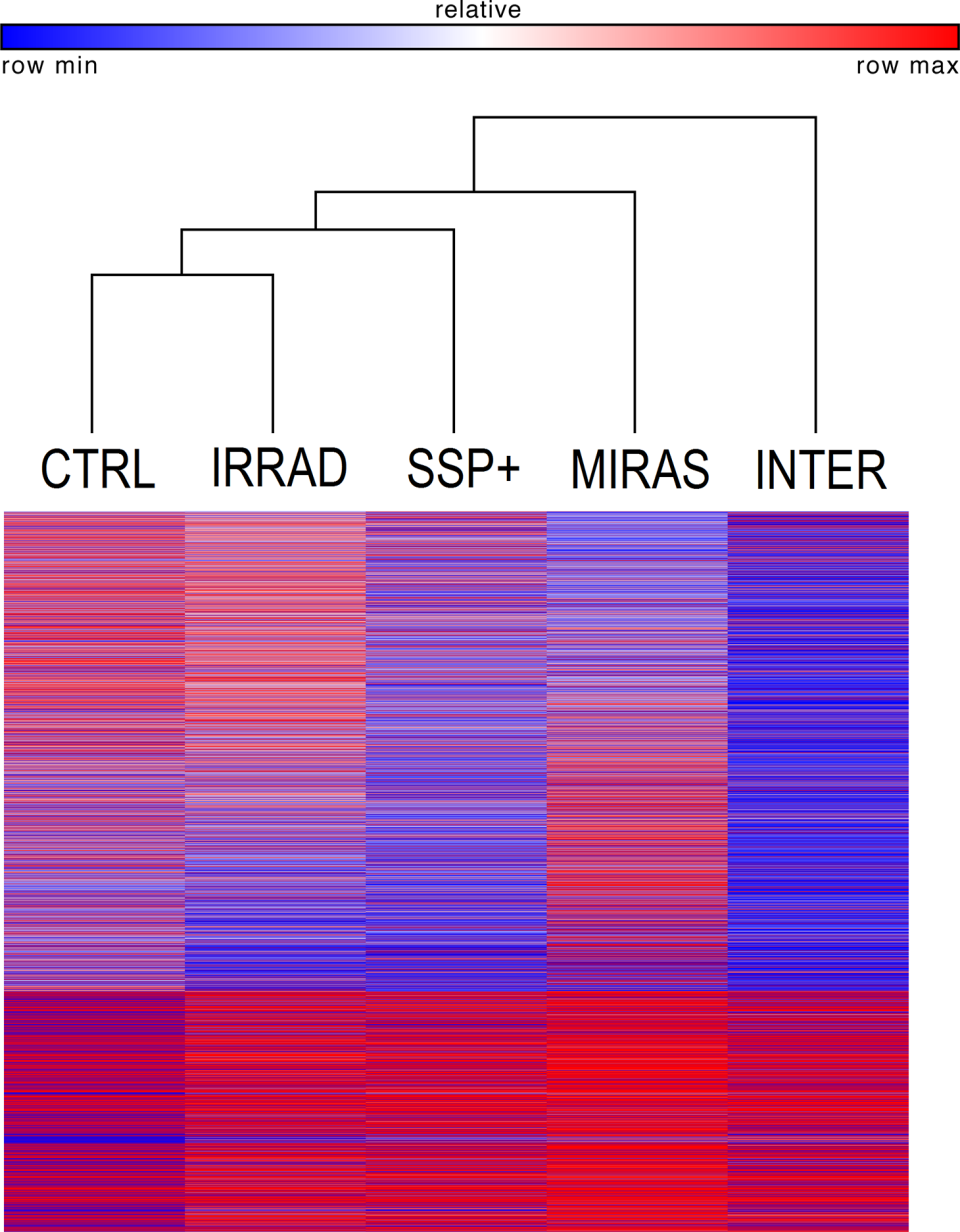
Hierarchical clustering of the five experimental groups with 20,803 mRNAs, analyzed by Spearman rank correlation and average linkage. CTRL = Control; IRRAD = Irradiated; SSP+ = SSP+; MIRAS = Mirasol; INTER = Intercept.

We performed hierarchical clustering of all five groups with 20,803 genes analyzed by using Spearman rank correlation and average linkage (Fig 3). The dendrogram shows that irradiated platelets co-cluster with control platelets, and that Intercept-treated platelets form a separate cluster distant from control platelets. Apparently, the row color for Intercept transcripts indicates a global divergence of gene expression leaning towards downregulation, as compared to the control.

We examined the possibility that gender might be a confounding variable. Our previous study [19], reported a panel of 18 transcripts that were differentially expressed between sexes (2 males and 1 female) in human platelets. In the current study, however, we found no significant differences in mRNA expression levels that could be attributed to gender when we compared the three males and the single female in the control group. A pairwise correlation analysis of the control group revealed congruity in gene expression between the sexes (S1 Fig). This study, therefore, did not find gender as a confounder of concern in the data analysis. The lack of conformity between the studies of Kissopoulou et al. [19] and the current in this context may, at least in part, be explained by the fact that the former employed analysis of total RNA, whereas the latter investigated a specified number of genes corresponding to approximately 20,800 RefSeq genes. For instance, as many as 9 of the 18 transcripts that were found to be differentially expressed between sexes in the study of Kissopoulou et al. [19] were not detected in this study as they were not included in the Ion AmpliSeq Expression panel or were expressed in low copy numbers and did not survive the stringent threshold filter 1/10,000 of ACTB.

### Differential expression (DE) analysis at gene level

**Fig 4:**
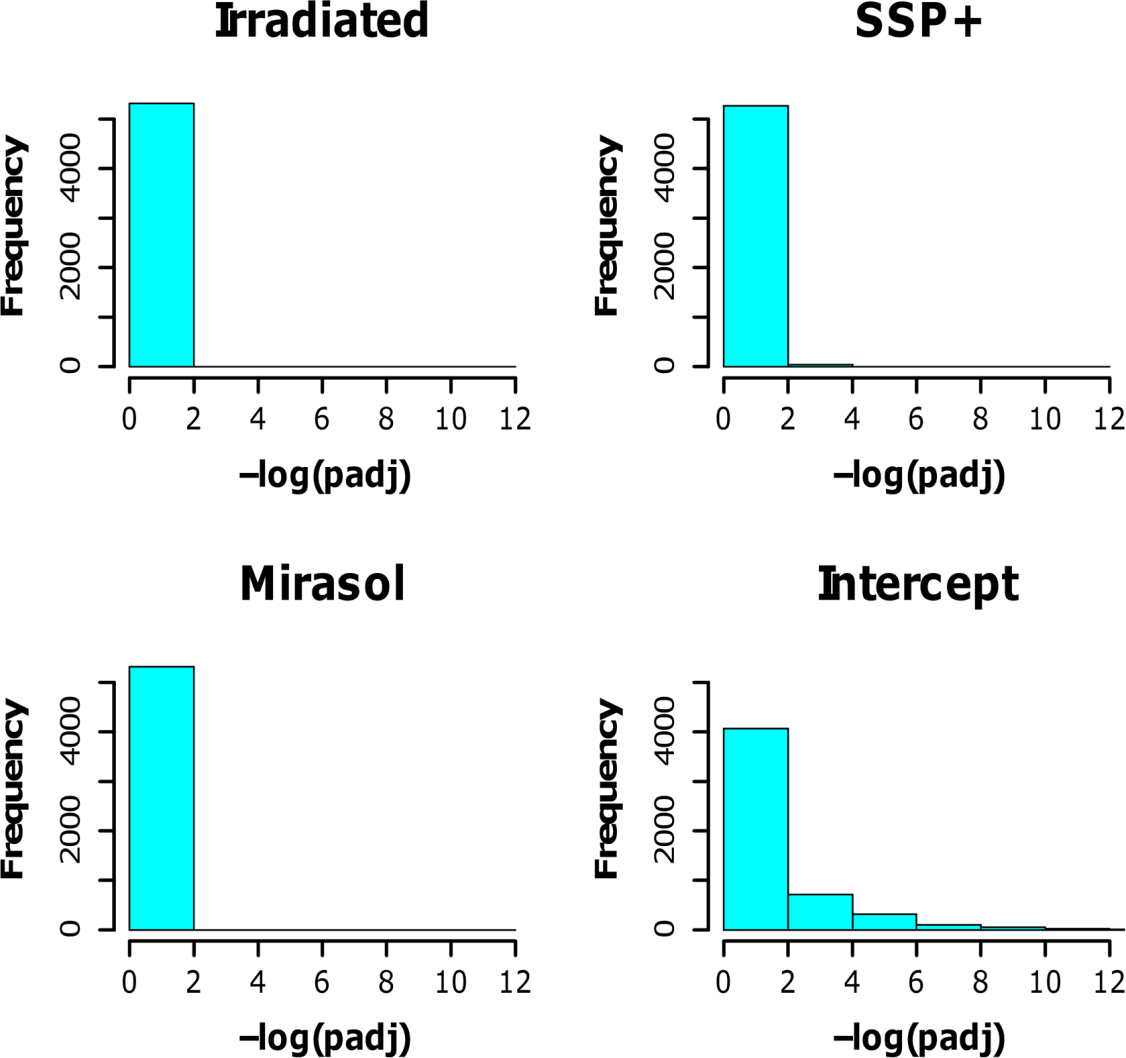
The adjusted p-values (padj) obtained from DESeq-analysis and corresponding frequencies of platelet mRNAs upon treatment for pathogen reduction. Differential platelet mRNA expression was calculated relative to the control group.

Complete DE tables can be found in S2 Table (irradiated), S3 Table (Mirasol), S4 Table (SSP+) and S5 Table (Intercept). We employed the DESeq software to analyze DE of mRNAs in the four treated platelet groups relative to the transcriptome of untreated control platelets. DE was expressed as log2FoldChange (FC) with corresponding p-value for each gene. Graphical representations depicting frequencies of adjusted p (padj)-values for all treatment groups are shown in Fig 4. This Figure shows the number of genes that were above the threshold 1/10,000 of β-actin expression and differentially expressed at specific levels of statistical significance.

In these mRNA DE analysis, we found that none of the > 5,300 platelet transcripts detected was differentially expressed in the irradiated platelets (all had padj-values of 1), compared to control platelets, indicating that gamma-irradiation has no impact on the platelet mRNA transcriptome (Fig 4). In addition, we found no evidence that Mirasol treatment of platelets entails a notable effect on the platelet mRNA transcriptome, as none of the observed changes in the Mirasol group reached statistical significance, when compared to the control group (Fig 4).

The mRNAs of 6 genes (*SVIP*, *EP300*, *PSIP1*, *TRIM58*, *SERTAD2 and FYTTD1*) were found to be deregulated upon SSP+ treatment of platelets, with fold changes reaching statistical significance (padj < 0.05). All of these transcripts were downregulated with fold changes ranging from -3 to -5. When the padj value was set at a more stringent threshold of <0.01, only three transcripts, *SVIP*, *EP300* and *PSIP1*, remained altered in the SSP+ group, with fold changes of -3.1, -4.9 and -3.5, respectively.

The treatment that exhibited the most striking effect on the platelet mRNA transcriptome was Intercept. Indeed, platelets treated with Intercept displayed an abnormal transcriptional profile compared with untreated, control platelets, with a substantial fraction of the detected mRNA transcripts being differentially expressed.

### Intercept significantly alters the platelet mRNA transcriptome

We identified 816 genes that were differentially expressed in Intercept-treated platelets, compared to control platelets, with a statistical significance level of padj < 0.05. Adding a FC≥2 threshold narrowed the list of differentially expressed mRNAs in Intercept-treated platelets to 400. We investigated the functional pathways, in which these genes may be involved on the Database for Annotation, Visualization and Integrated Discovery (DAVID) [22]. The network data provided by DAVID presented 7 statistically significant annotation clusters, most of which represent likely platelet pathways in membrane function, transport, metabolism and structure (Table 1). Of these 400 most significantly altered transcripts in Intercept-treated platelets, 302 were downregulated, suggesting a reduction of mRNA levels that can be attributed to Intercept-treatment. Apart from these downregulated mRNAs, 98 other transcripts were found to be differentially upregulated in Intercept-treated platelets. The mechanisms underlying the increase in the level of these platelet mRNAs induced by Intercept remain unclear, as platelets lack de novo genomic DNA transcription. However, we cannot exclude the possibility that these mRNAs exhibit an apparent increase because they may represent the least downregulated among all mRNAs. This might be possible since we used the same amount of RNA among all five groups to perform the DE RNA-Seq analysis (mRNAs that are downregulated leave room for other mRNAs when sampling a fixed amount of RNA).

**Table 1:** Functional annotation clusters generated by DAVID tools for the most significant (padj<0.05) differentially expressed (FC≥2) mRNAs in platelets treated with the Intercept pathogen reduction system.

Term	Functional Classification	Annotation Cluster	Count	P_Value	Benjamini
GOTERM_CC_FAT	Golgi apparatus	1	41	2.6E-06	2.5E-04
SP_PIR_KEYWORDS	ER-golgi transport	1	11	4.5E-06	4.5E-04
SP_PIR_KEYWORDS	Golgi apparatus	1	29	1.2E-05	6.9E-04
SP_PIR_KEYWORDS	Protein transport	1	25	2.8E-05	1.4E-03
SP_PIR_KEYWORDS	Transport	1	52	7.5E-04	2.3E-02
GOTERM_CC_FAT	Membrane-enclosed lumen	2	70	1.1E-06	2.0E-04
GOTERM_CC_FAT	Organelle lumen	2	69	1.1E-06	1.4E-04
GOTERM_CC_FAT	Intracellular organelle lumen	2	66	4.4E-06	3.4E-04
GOTERM_CC_FAT	Non-membrane-bounded organelle	2	86	8.1E-06	5.1E-04
GOTERM_CC_FAT	Intracellular non-membrane-bounded organelle	2	86	8.1E-06	5.1E-04
UP_SEQ_FEATURE	Nucleotide phosphate-binding region:ATP	3	38	6.0E-05	3.7E-02
SP_PIR_KEYWORDS	ATP-binding	4	50	9.4E-06	7.6E-04
SP_PIR_KEYWORDS	Nucleotide-binding	4	59	1.2E-05	7.7E-04
UP_SEQ_FEATURE	Nucleotide phosphate-binding region:ATP	4	38	6.0E-05	3.7E-02
GOTERM_CC_FAT	Cytoskeleton	5	49	3.4E-04	1.3E-02
SP_PIR_KEYWORDS	ER-golgi transport	6	11	4.5E-06	4.5E-04
SP_PIR_KEYWORDS	Metal-binding	7	80	1.7E-03	4.7E-02

**Fig 5:**
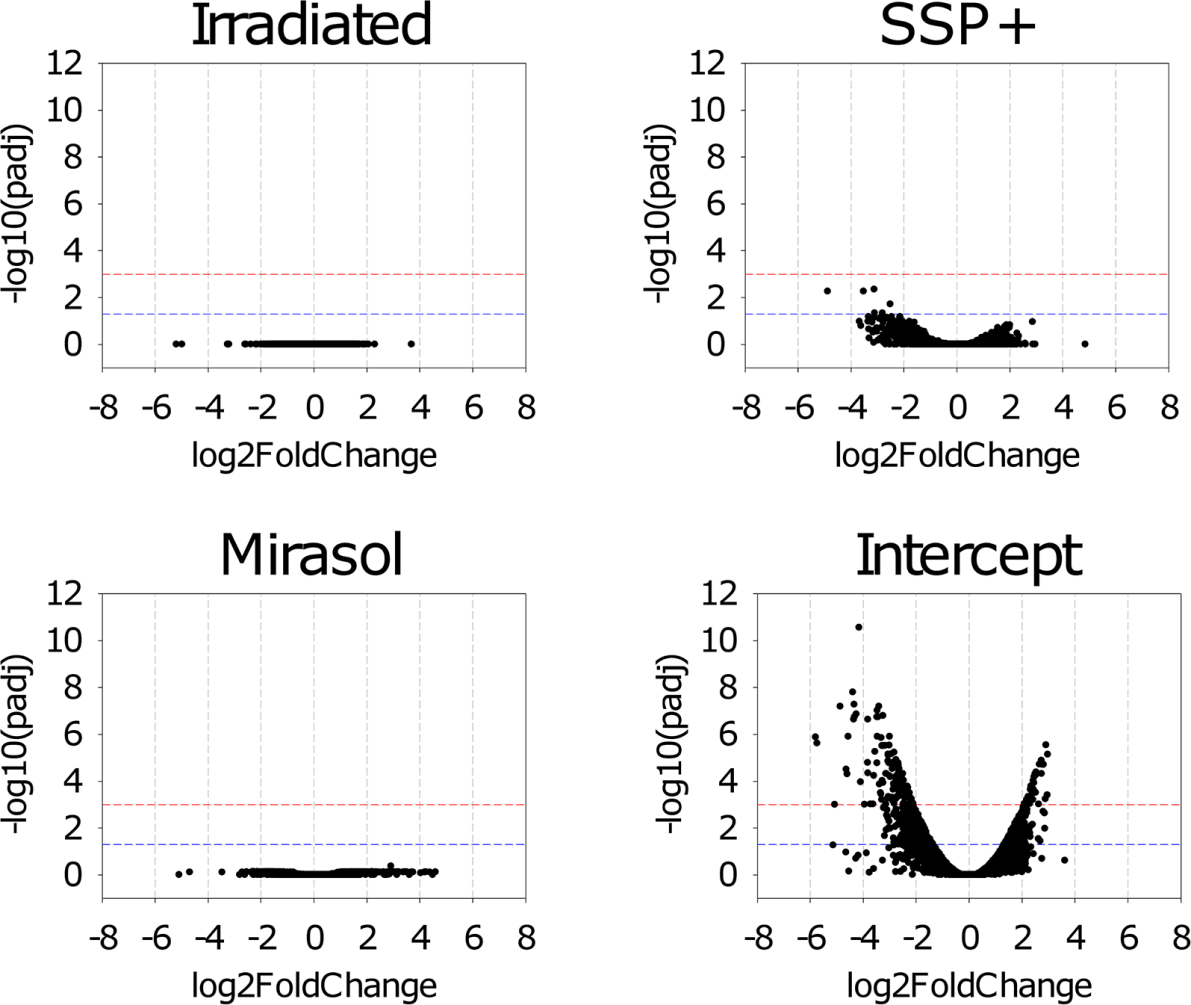
The fold changes (in log2) relative to the control group and the corresponding adjusted p-values (padj) are depicted for the pathogen reduction treatment groups. Blue and red horizontal dotted lines indicate padj thresholds at 0.05 and 0.001, respectively.

A Volcano plot, in which the padj-values for all detected transcripts in the four treatment groups are plotted as a function of log2FoldChanges, shows how considerable is the impact of Intercept on the platelet mRNA transcriptome (Fig 5). Only Intercept-treated platelet mRNAs displayed significant FC when the threshold of padj-value was set at <0.001, as 147 genes were differentially expressed by ≥ 2 fold in the Intercept group.

To identify DE hotspots (i.e. the extreme points of differentially expressed genes) in Intercept-treated platelets, we further increased the threshold of FC to >4.0 at padj < 0.001. This generated a panel of 13 highly significant and markedly downregulated genes (Table 2). There were no upregulated genes in this cluster. The most statistically significant gene in this list, *KCNA3* (padj = 2.8x10^-11^), encodes the potassium voltage-gated channel, shaker-related subfamily, member 3 (Kv1.3). Interestingly, McCloskey et al. [23] recently reported that Kv1.3 forms the voltage-gated K+ channel of platelets and megakaryocytes, is responsible for the major K+ conductance and resting potential of platelets, and influences the number of circulating platelets.

**Table 2:** Thirteen (13) platelet mRNAs that were found to be downregulated by more than 4 fold (p-value < 0.001) upon treatment with Intercept, ranked in order of DE significance by padj values.

Gene symbol	Gene name	Chromosomal location	FC	log2FC	Pval	Padj
KCNA3	potassium voltage-gated channel, shaker-related subfamily, member 3	1p13.3	0.057	-4.1	1.9e^-15^	2.8e^-11^
NCOA3	nuclear receptor coactivator 3	20q12	0.048	-4.4	2.2e^-12^	1.6e^-08^
DDB1	damage-specific DNA binding protein 1, 127kDa	11q12-q13	0.050	-4.3	1.1e^-11^	5.4e^-08^
DDX41	DEAD (Asp-Glu-Ala-Asp) box polypeptide 41	5q35.3	0.035	-4.9	2.3e^-11^	6.6e^-08^
LY6G6E	lymphocyte antigen 6 complex, locus G6E (pseudogene)	6p21.3	0.053	-4.3	6.7e^-11^	1.4e^-07^
AKT2	v-akt murine thymoma viral oncogene homolog 2	19q13.1-q13.2	0.050	-4.3	1.4e^-10^	1.9e^-07^
RBL2	retinoblastoma-like 2	16q12.2	0.049	-4.3	2.3e^-10^	2.4e^-07^
SETD5	SET domain containing 5	3p25.3	0.042	-4.6	1.4e^-09^	1.3e^-06^
DAXX	death-domain associated protein	6p21.3	0.018	-5.8	1.8e^-09^	1.4e^-06^
TSPYL4	TSPY-like 4	6q22.1	0.019	-5.7	3.5e^-09^	2.5e^-06^
CNNM4	cyclin and CBS domain divalent metal cation transport mediator 4	2q11.2	0.040	-4.6	1.2e^-07^	3.2e^-05^
PRRC2A	proline-rich coiled-coil 2A	6p21.3	0.041	-4.6	2.2e^-07^	5.1e^-05^
GGA2	golgi-associated, gamma adaptin ear containing, ARF binding protein 2	16p12	0.059	-4.1	5.9e^-07^	1.1e^-04^

FC = Fold change; pval = p-value; padj = adjusted p-value

These results indicate that the Intercept system, which is either used or considered to be implemented for pathogen reduction of blood components by blood banks, markedly alters the platelet mRNA transcriptome, which may negatively impact the platelets’ response and function involving de novo mRNA translation into bioactive effector proteins.

### Integrated miRNA and mRNA analysis revealed network of multiple correlated miRNA-mRNA pairs

To investigate the existence of possible mRNA-miRNA correlations among the stored platelet groups, we employed the MAGIA analysis tool with miRNA target prediction, followed by a regression analysis. The expression profiles for mRNAs that were differentially expressed in Intercept platelets with a FC≥2 and a p<0.01 were included in the analyses. For miRNAs, expression profiles of 11 miRNAs previously analyzed in the same samples [11] were employed. This analysis revealed the existence of multiple correlations between differentially expressed mRNAs and miRNAs in platelets. Specifically, 20 inverse (Table 3) and 20 positive (Table 4) correlations were identified between 5 of the 11 miRNAs investigated and several mRNAs that are differentially expressed in Intercept-treated platelets.

**Table 3:** Inverse miRNA-mRNA correlations found in platelets treated with Intercept. Genes differentially overexpressed at p < 0.01 were included in the analysis. All correlations were statistically significant at p < 0.05.

Ensembl gene ID	Symbol	miRNA	Correlation
ENSG00000185262	FAM100B	hsa-miR-484	-1.0
ENSG00000100325	ASCC2	hsa-miR-484	-0.9
ENSG00000182087	C19orf6	hsa-miR-24	-0.9
ENSG00000182087	C19orf6	hsa-miR-484	-0.9
ENSG00000137343	ATAT1	hsa-miR-484	-0.9
ENSG00000119004	CYP20A1	hsa-miR-17	-0.9
ENSG00000119004	CYP20A1	hsa-miR-484	-0.9
ENSG00000158161	EYA3	hsa-miR-24	-0.9
ENSG00000186642	PDE2A	hsa-miR-24	-0.9
ENSG00000122741	DCAF10	hsa-miR-484	-0.9
ENSG00000180353	HCLS1	hsa-miR-484	-0.7
ENSG00000143851	PTPN7	hsa-miR-24	-0.7
ENSG00000143851	PTPN7	hsa-miR-484	-0.7
ENSG00000136868	SLC31A1	hsa-miR-17	-0.7
ENSG00000136868	SLC31A1	hsa-miR-24	-0.7
ENSG00000136868	SLC31A1	hsa-miR-484	-0.7
ENSG00000161888	SPC24	hsa-miR-484	-0.7
ENSG00000166548	TK2	hsa-miR-24	-0.7
ENSG00000166548	TK2	hsa-miR-484	-0.7
ENSG00000185651	UBE2L3	hsa-miR-484	-0.7

**Table 4:** Positive miRNA-mRNA correlations found in platelets treated with Intercept. Genes differentially downregulated at p < 0.01 were included in the analysis. All correlations were statistically significant at p < 0.05.

Ensembl gene ID	Gene symbol	microRNA	Correlation
ENSG00000204427	ABHD16A	hsa-miR-24	1.0
ENSG00000204427	ABHD16A	hsa-miR-484	1.0
ENSG00000047644	WWC3	hsa-miR-484	1.0
ENSG00000130052	STARD8	hsa-miR-484	0.9
ENSG00000168488	ATXN2L	hsa-miR-484	0.7
ENSG00000128578	FAM40B	hsa-let-7e	0.7
ENSG00000100030	MAPK1	hsa-miR-106a	0.7
ENSG00000100030	MAPK1	hsa-miR-17	0.7
ENSG00000100030	MAPK1	hsa-miR-24	0.7
ENSG00000100030	MAPK1	hsa-miR-484	0.7
ENSG00000149480	MTA2	hsa-miR-484	0.7
ENSG00000163590	PPM1L	hsa-miR-24	0.7
ENSG00000163590	PPM1L	hsa-miR-484	0.7
ENSG00000158352	SHROOM4	hsa-miR-24	0.7
ENSG00000158352	SHROOM4	hsa-miR-484	0.7
ENSG00000134668	SPOCD1	hsa-miR-24	0.7
ENSG00000134668	SPOCD1	hsa-miR-484	0.7
ENSG00000182253	SYNM	hsa-miR-484	0.7
ENSG00000139722	VPS37B	hsa-miR-24	0.7
ENSG00000139722	VPS37B	hsa-miR-484	0.7

**Fig 6:**
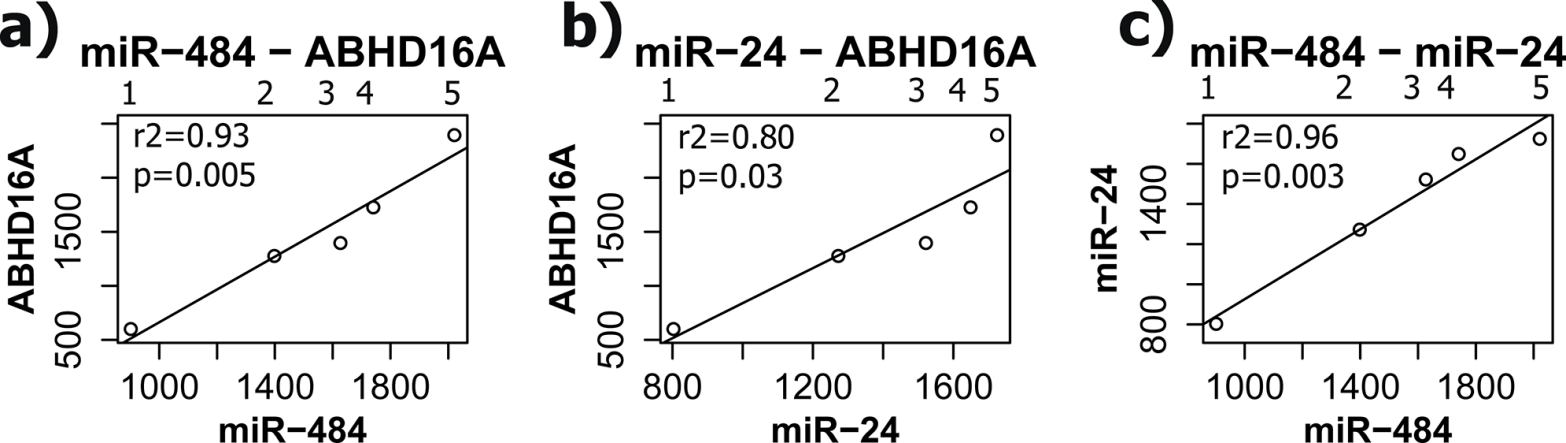
Positive correlations of miRNA-mRNA (a–b) and miRNA-miRNA (c) pairs in platelet concentrates (PCs) treated with different pathogen reduction systems and the control. Correlations are shown between miR-484 and *ABHD16A* (a), miR-24 and *ABHD16A* (b) and between miR-24 and miR-484 (c). On the x-axis, the relative expression of each miRNA is shown, whereas the normalized expression of mRNA is shown on the y-axis: mRNA-expression = (number of reads/(1/10,000 of ACTB reads)*100. R-squared (r2) and regression significance (p) are shown above the trend line. Position of each platelet group is shown above the diagram: 1 = Intercept; 2 = Irradiated; 3 = Mirasol; 4 = SSP+; 5 = Control.

**Fig 7:**
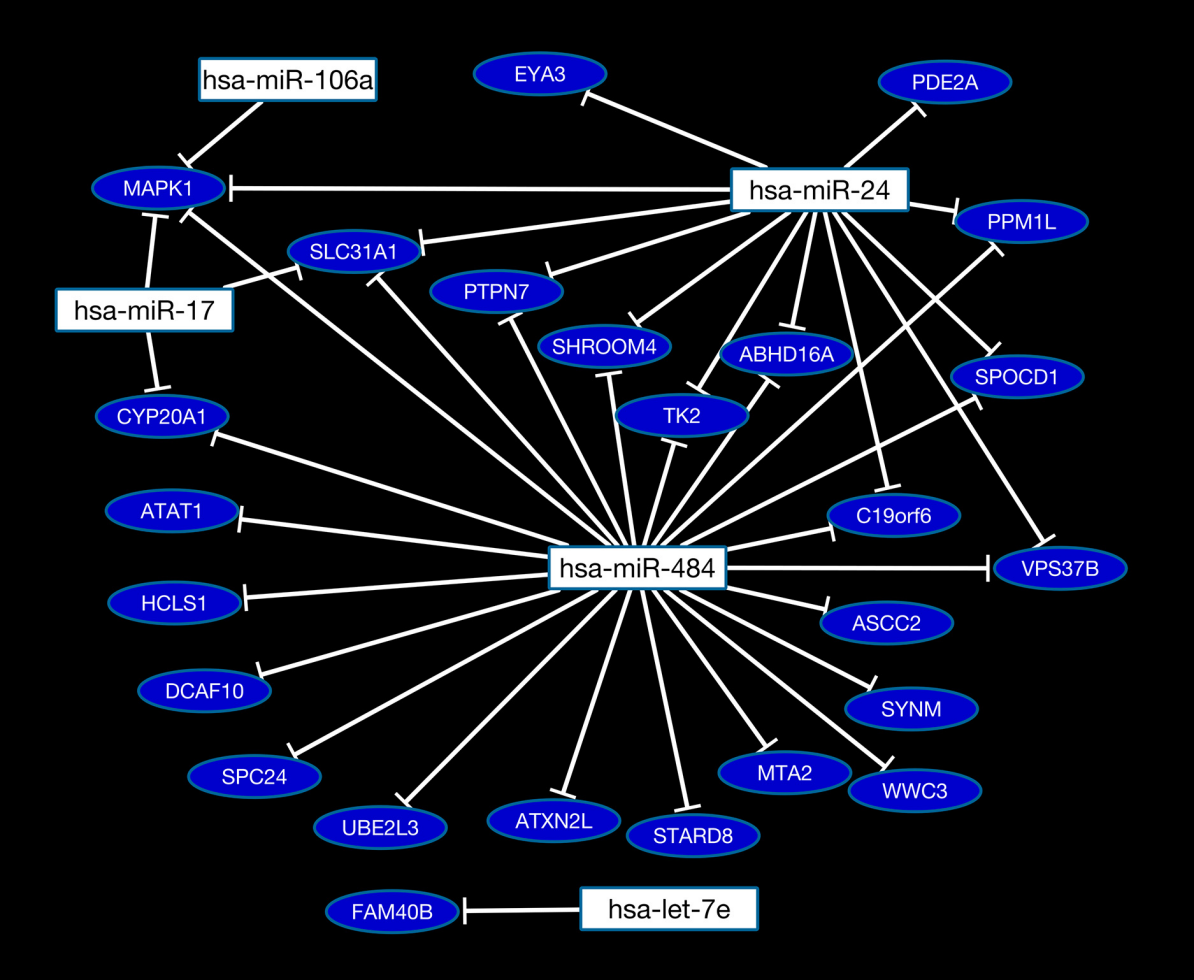
A miRNA-mRNA network illustrating the interactions between downregulated mRNAs (p<0.01) and miRNAs (p<0.05) in platelets treated with Intercept. The interaction network was constructed by combining miRNA target prediction using MiRanda and PITA tools with experimentally measured expression levels of mRNAs and miRNAs in the same samples.

To infer the most pronounced miRNA-mRNA relationships in Intercept platelets, we increased the stringency of the analysis and considered only those mRNAs that were differentially expressed below the p-value threshold of 0.001. From this analysis, 2 different miRNA-mRNA pairs, both involving *ABHD16A* (miR-484•*ABHD16A* and miR-24• *ABHD16A*) were positively correlated that were found to be statistically significant (p<0.05) after performing regression analysis (Fig 6A–6B). Interestingly, miR-484 was previously found to be significantly downregulated in Intercept platelets [11], and its positive correlation with *ABHD16A* mRNA that is also downregulated in Intercept platelets may suggest a regulatory role of miR-484 for this gene. Similarly, miR-24 also tended to be downregulated in Intercept platelets by nearly two fold (albeit not reaching statistical significance), as we reported previously [11]. Moreover, the two miRNAs (miR-484 and miR-24) were found to be strongly correlated with each other (Fig 6C), suggesting that they depart from the Intercept-treated platelets along with their predicted target mRNAs. A complete network of all identified miRNA-mRNA interaction pairs is shown in Fig 7. This network shows miRNAs associated with the most significantly (p<0.01) downregulated mRNAs in Intercept platelets.

## Discussion

Current PR systems are designed to inactivate the infectious pathogens that contaminate blood components by cross-linking their nucleic acids and impairing their function. This strategy is effective in preventing transfusion-transmitted infections, but does not take into account the fact that human platelets may require functional mRNAs for *de novo* synthesis of proteins that will mediate their response to the environmental cues to which they are exposed. Despite their anucleate nature, platelets harbor a diverse repertoire of genetic materials that is rich, versatile and functional [14, 24]. Indeed, accumulating evidences indicate that human platelets (i) contain a vast repertoire of mRNAs that reflects and determines their function (reviewed in [25]), (ii) have pre-mRNAs that can be spliced into mature and functional mRNAs [24], (iii) perform *de novo* translation of certain mRNAs into proteins upon their activation [5], (iv) are equipped with the essential components of the translational machinery [1, 3, 4], and (v) contain all major classes of noncoding RNAs, including microRNAs that were found to be functional in regulating mRNA translation [25, 26]. These evidences strongly suggest that nucleic acids play an important role in platelet function, so that alteration in the level or function of nucleic acids may lead to deregulation of platelet function.

We previously reported that Intercept-treatment of stored platelets downregulated 6 of 11 microRNAs and 2 of 3 mRNAs that were analyzed by qPCR [11]. Here, we extended these findings and demonstrated that, among the PR systems under study, Intercept is the approach that most profoundly altered the transcriptional landscape of platelets, compared to untreated, control platelets stored under normal blood bank conditions.

Consistent with our previous report [11], the SSP+ additive solution have some effects on the platelet mRNA transcript profile, as 6 genes were differentially expressed at p<0.05. Because Intercept involves storage of platelets in additive solution (SSP+ in this study), the effects that we observed in the Intercept group may be partially explained by SSP+. However, when comparing the number of genes that are affected by Intercept (816 genes) versus SSP+ (6 genes) at the p<0.05 threshold, it is clear that the amotosalen + ultraviolet-A light treatment, rather than the SSP+, is responsible for most of the adverse effects of Intercept on the platelet mRNA transcriptome. Especially since only Intercept-treated platelets displayed altered expression of genes, 147 to be precise, at p<0.001. We previously showed that Intercept-treatment of stored platelets was also associated with (i) a reduction in the expression level of 6 microRNAs, (ii) platelet activation, (iii) an impaired platelet aggregation response to adenosine diphosphate (ADP), (iv) reduction of the mean platelet volume, and (v) the release of microparticles containing microRNAs [11]. SSP+ also induced similar effects on platelet miRNA levels, albeit to a much lesser extent [11], along a trend similar to that we are reporting for mRNAs. We cannot exclude that the negative impact of Intercept on the platelet transcriptome that we observed here most likely is maintained during the entire storage time and may evolve like that of microRNAs, which we documented for up to 7 days of storage under blood bank conditions. It is also important to underline that platelet mRNA levels can hardly recover in the absence of genomic DNA transcription, a process that is absent in the anucleate platelets.

Our mRNA list for the control group correlated well with the dataset reported by Londin et al. [17], despite the different protocols applied in each study. Nearly 80% of all of our detected mRNAs in the control group could be identified in the list reported by Londin et al. [17]. The remaining ~20% unmatched transcripts may be explained by the different sequencing protocols applied in each study and perhaps differences in genetic background, ethnicity, age and lifestyle of the recruited blood donors as well as the differing geographical locations where each study was performed.

It is interesting to note that 2 miRNA-mRNA pairs were significantly and positively correlated among the 5 groups of stored platelets, which is suggestive of their association in stored human platelets. A panel of only 11 miRNAs was included in the analysis and it is therefore reasonable to believe that other similar pairs of uninvestigated miRNAs and their mRNA targets may exist in the samples analyzed. In that context, it is tempting to speculate that Intercept-treatment of stored platelets may induce the departure of individual nucleic acid molecules as well as of miRNAs bound to their mRNA targets. Such a scenario would explain the positive correlation and downregulation of miR-484 and its predicted target mRNA (*ABHD16A*) upon Intercept-treatment of stored platelets. Whether Intercept induces the release of platelet mRNAs through microparticles, like miRNAs, as we suggested previously, remains to be investigated. As well, the idea that Intercept may facilitate the uptake of external nucleic acids by the stored platelets may be worth pursuing.

## Conclusions

The results of this study indicate that Intercept-treatment markedly and unequivocally modify the platelet transcriptome, which may underlie the corresponding alterations in platelet activation and function that we reported previously. These findings should enlighten and encourage authorities worldwide to be more vigilant when (i) analyzing the adverse effects associated with Intercept, (ii) evaluating the perspective of implementing the use of new PR systems, and (iii) weighing the risk/benefits, or the pros and cons, of implementing the use of certain PR systems for the prevention of transfusion-transmitted infections. We believe that modifications of current technologies and/or the development of approaches targeting pathogens more specifically, while sparing platelet nucleic acids level and function, would be highly desirable and should be encouraged.

## Supporting Information

S1 Fig Pairwise correlation analysis in platelet mRNA expression of the control group (3 males and 1 female). R-squared values are shown above each plot.
(TIFF)

S1 Table The 50 most highly abundant mRNAs in each platelet group using mean mRNA expressions (n = 4 per group). * Transcripts that were not found in the top 50 of the other groups.
(TXT)

S2 Table Differential expression data for irradiated platelets. (TXT)

S3 Table Differential expression data for platelets treated with Mirasol pathogen reduction. (TXT)

S4 Table Differential expression data for platelets stored in SSP+ additive solution. (TXT)

S5 Table Differential expression data for platelets treated with Intercept pathogen reduction. (TXT)
