# Supplementary figures and images for: Differential Expression Analysis by RNA-Seq Reveals Perturbations in the Platelet mRNA Transcriptome Triggered by Pathogen Reduction Systems
Source: PLoS One. 2015 Jul 14;10(7):e0133070. doi: 10.1371/journal.pone.0133070 (PMC4501785; doi:10.1371/journal.pone.0133070)

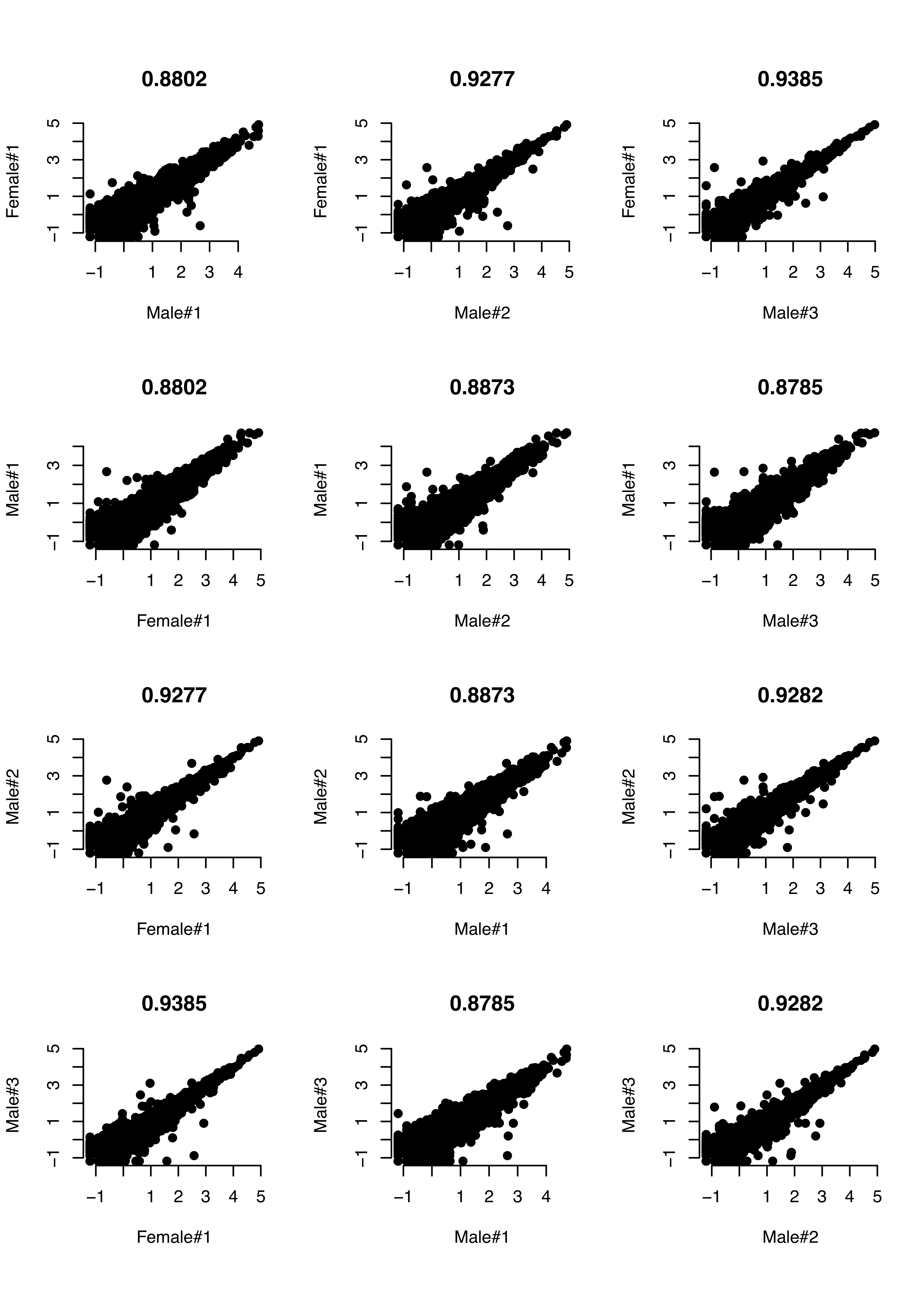

Supplement: S1 Fig — R-squared values are shown above each plot. (TIFF) [file pone.0133070.s001.tiff]
